# Role of Plasma Gelsolin Protein in the Final Stage of Erythropoiesis and in Correction of Erythroid Dysplasia In Vitro

**DOI:** 10.3390/ijms21197132

**Published:** 2020-09-27

**Authors:** So Yeon Han, Eun Mi Lee, Suyeon Kim, Amy M. Kwon, Eun Jung Baek

**Affiliations:** 1 Department of Laboratory Medicine, College of Medicine, Hanyang University, Seoul 04763, Korea; soyeon806@gmail.com (S.Y.H.); rainbow1114@hanmail.net (S.K.); 2 Department of Translational Medicine, Graduate School of Biomedical Science and Engineering, Hanyang University, Seoul 04763, Korea; ghlflek@gmail.com; 3 Biostatistical Consulting and Research Laboratory, Medical Research Collaborating Center, Industry-University Cooperation Foundation, Hanyang University, Seoul 04763, Korea; amykwon@hanyang.ac.kr

**Keywords:** gelsolin, erythropoiesis, in vitro cell culture, erythrocytes, myelodysplastic syndrome

## Abstract

Gelsolin, an actin-remodeling protein, is involved in cell motility, cytoskeletal remodeling, and cytokinesis and is abnormally expressed in many cancers. Recently, human recombinant plasma gelsolin protein (pGSN) was reported to have important roles in cell cycle and maturation of primary erythroblasts. However, the role of human plasma gelsolin in late stage erythroblasts prior to enucleation and putative clinical relevance in patients with myelodysplastic syndrome (MDS) and hemato-oncologic diseases have not been reported. Polychromatic and orthochromatic erythroblasts differentiated from human cord blood CD34+ cells, and human bone marrow (BM) cells derived from patients with MDS, were cultured in serum-free medium containing pGSN. Effects of pGSN on mitochondria, erythroid dysplasia, and enucleation were assessed in cellular and transcriptional levels. With pGSN treatment, terminal maturation at the stage of poly- and ortho-chromatic erythroblasts was enhanced, with higher numbers of orthochromatic erythroblasts and enucleated red blood cells (RBCs). pGSN also significantly decreased dysplastic features of cell morphology. Moreover, we found that patients with MDS with multi-lineage dysplasia or with excess blasts-1 showed significantly decreased expression of gelsolin mRNA (*GSN*) in their peripheral blood. When BM erythroblasts of MDS patients were cultured with pGSN, levels of mRNA transcripts related to terminal erythropoiesis and enucleation were markedly increased, with significantly decreased erythroid dysplasia. Moreover, pGSN treatment enhanced mitochondrial transmembrane potential that is unregulated in MDS and cultured cells. Our findings demonstrate a key role for plasma gelsolin in erythropoiesis and in gelsolin-depleted MDS patients, and raises the possibility that pGSN administration may promote erythropoiesis in erythroid dysplasia.

## 1. Introduction

Recent studies have elucidated the complex mechanisms underlying late-stage erythropoiesis by examining the in vitro generation of stem cell-derived red blood cells (RBCs). However, there are problems recapitulating this process in vitro, the main issues being delayed maturation, inefficient enucleation, low viability, dysregulated apoptosis, and dysplastic cell features, such as variable cell size and multi-nucleation [1,2,3,4]. These effects are thought to be due to the absence of certain stimulatory factors and signals, and to dysregulated autophagy. Interestingly, these phenomena are not confined to in vitro conditions, but are very similar to those found in patients with myelodysplastic syndrome (MDS) [5,6]. In MDS, hematopoietic stem cells have defects in maturing into functional blood cells showing unbalanced cell cycle between nuclei and cytoplasm, irregular cell size, and dysregulated apoptosis [7].

Gelsolin is an 83 kDa single-chain protein that controls actin polymerization and depolymerization via Ca^2+^ and anti-apoptotic proteins in humans [8,9]. There are three gelsolin isoforms encoded by a single *GSN* gene resulting from alternative splicing: a plasma-type isoform and two cytoplasmic forms (cGSN), which are related to actin modulation. Human plasma gelsolin can cross cytoplasmic membranes into the bloodstream owing to the presence of a 25 amino acid N-terminal secretory signal sequence [10]. The functions of plasma gelsolin and cGSN have been suggested to be similar [11]. Both plasma gelsolin and cGSN are involved in severing filamentous actin (F-actin) [12,13]. This actin severing process is essential for the dynamic changes to the actin cytoskeleton occurring in many cell types, including macrophages [14]. In erythroblasts, F-actin is critical for enucleation, and is involved in a spectrin-actin-based membrane network during erythropoiesis [15].

We recently demonstrated that plasma gelsolin protein (pGSN) can enter immature erythroid cells at the stage of basophilic erythroblasts and reduces dysplastic changes by recovering the blocked G2/M cell cycle [16]. However, the roles of pGSN have not been known in late stage erythroblasts (polychromatic and orthochromatic erythroblasts) before enucleation. These mature cells show marked changes from their precursors by preparing cell cycle exit, cell skeleton reorganization, different apoptotic signaling, and autophagic mitochondria [5,17]. Moreover, its relation with MDS and the plasma gelsolin levels in patients’ blood, and effects on patients’ dysplastic cells have not been studied yet.

Dysplastic erythropoiesis is one of the most important features of MDS, which involves clonal hematopoietic stem cell malignancies [18]. There have been many studies surrounding the mechanisms of abnormal erythropoiesis in MDS [7,18]. Moreover, actin filament defects are responsible for the erythroid dysplasia seen in erythroblasts of patients with MDS [19]. However, there are few studies involving human erythroid dysplasia pathophysiology during the terminal maturation phase due to lack of both proper animal models and erythroid cell culture systems.

We, therefore, hypothesized that the problems associated with in vitro terminal erythropoiesis and dysplastic erythropoiesis, such as poor cell viability, low levels of enucleation, the dysplastic features of nuclei and cytoplasm, and the mitochondrial abnormalities, were partially due to insufficient gelsolin, which is normally supplied to late erythroid cells by macrophages and mesenchymal stem cells. In this study, we treated erythroid cells with recombinant human plasma gelsolin (pGSN) by adding it to cell culture medium.

Here, we show novel roles of pGSN during human terminal erythropoiesis and dysplastic erythropoiesis. The *GSN* mRNA levels were markedly low in peripheral blood (PB) from patients with some MDS subtypes and other hemato-oncologic diseases. Furthermore, when bone marrow (BM) cells derived from patients with MDS were cultured in the presence of pGSN, the erythroid dysplasia was improved, suggesting its therapeutic potential for MDS patients.

## 2. Results

### 2.1. pGSN Accelerates In Vitro Terminal Erythropoiesis and Corrects Erythroid Dysplasia

**Figure 1:**
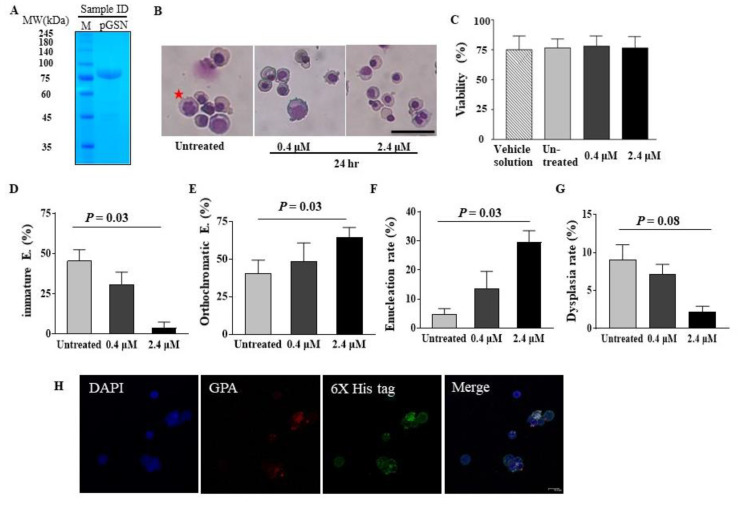
pGSN stimulates terminal maturation of cord blood (CB)-derived late erythroblasts. (**A**) The purity of recombinant human plasma gelsolin (pGSN) was assessed by SDS-polyacrylamide gel electrophoresis (PAGE) followed by staining with Coomassie Brilliant Blue. (M, protein molecular weight marker) (**B**) CB-derived late stage erythroblasts, consisting of more than 30% orthochromatic erythroblasts, were treated with pGSN for 24 h. With pGSN, most cells matured to orthochromatic erythroblasts and red blood cells (RBCs). Representative images of 3 CB cases are shown. Red star, dysplastic erythroblast. Scale bar = 50 µm, Wright-Giemsa staining. (**C**) The viability of late stage erythroblasts after treatment with pGSN or the same amount of vehicle solution for 24 h. (**D**) The proportion of immature erythroblasts (E.) consisted with basophilic erythroblasts and polychromatic erythroblasts, (**E**) orthochromatic erythroblasts, (**F**) enucleation rate, and (**G**) dysplastic erythroblast rate were compared after treatment with pGSN for 24 h. More than 200 cells scored in 7 independent fields by two experts. Data are mean ± standard error of the mean. (*n* = 3) (**H**) Orthochromatic erythroblasts were isolated at day 16 of culture and treated with His-tagged pGSN for 24 h. Cells were stained with 4,6-diamidino-2-phenylindole dihydrocloride (DAPI) and anti-His and analyzed by confocal microscopy (Blue, DAPI; Red, glycophorin A (GPA); Green, His-Tag). Scale bar, 10 µm.

To evaluate whether pGSN enhances terminal maturation and decreases erythroid dysplasia of in vitro cultured late stage erythroid cells, CB CD34+ cells were differentiated to polychromatic or orthochromatic erythroblasts until day 15. After the purity of recombinant pGSN protein was verified (Figure 1A), pGSN was added to the serum-free culture medium when the percentage of orthochromatic erythroblasts exceeded 30% (Figure 1B). While late stage erythroblasts usually show low viability in serum-free and stromal cell-free culture conditions, the cell viability was similar among group of untreated, vehicle solution treated, and pGSN conditions, ruling out the toxicity of pGSN on erythroid cells (Figure 1C). After 24 h of culture with pGSN, the percentage of immature basophilic/polychromatic erythroblasts was significantly lower than in the control (Figure 1D) and the percentage of mature orthochromatic erythroblasts was significantly increased compared to that in controls, showing that pGSN enhances in vitro erythroid maturation (Figure 1E). Enucleation rate was significantly elevated at higher concentrations of pGSN than that in controls (Figure 1F). Importantly, the dysplasia rate in erythroid cells was significantly decreased at the higher pGSN concentration, suggesting that pGSN could correct erythroid dysplasia (Figure 1G).When the purity and maturation stages were evaluated using erythroid cell specific markers (GPA and CD71) by flow cytometry, the erythroid lineage was very high up to 99.9%, demonstrating the high purity of erythroid cells and terminally mature stages (Figure S1). To confirm the pGSN can enter the polychromatic and orthochromatic erythroblasts around the enucleation stage, orthochromatic erythroblasts were purely isolated using gradient centrifugation by Percoll and then cultured with pGSN. By confocal images, we could observe His-tagged pGSN inside cells (Figure 1H). Taken together, these findings suggest that pGSN can partially overcome defective cytokinesis during terminal erythropoiesis, and stimulate terminal cell maturation, and enucleation.

To rule out that pGSN function is related to extracellular LPA which is secreted by monocyte/macrophages and known to bind pGSN and enhance erythropoiesis [20,21], LPA concentrations in raw media (Stemline II, Sigma) and conditioned media cultured with erythroid cells for 24 h were measured. The amount of standard LPA as control was measured as 17 ng/mL, but the other sample did not show any measurable peak, therefore excluding extracellular effects of pGSN via LPA on erythroid cells (Figure S2).

### 2.2. Effects of pGSN on Cell Size, Nuclear Size, Actin Skeleton, and Dysplasia of Late Stage Erythroblasts

**Figure 2:**
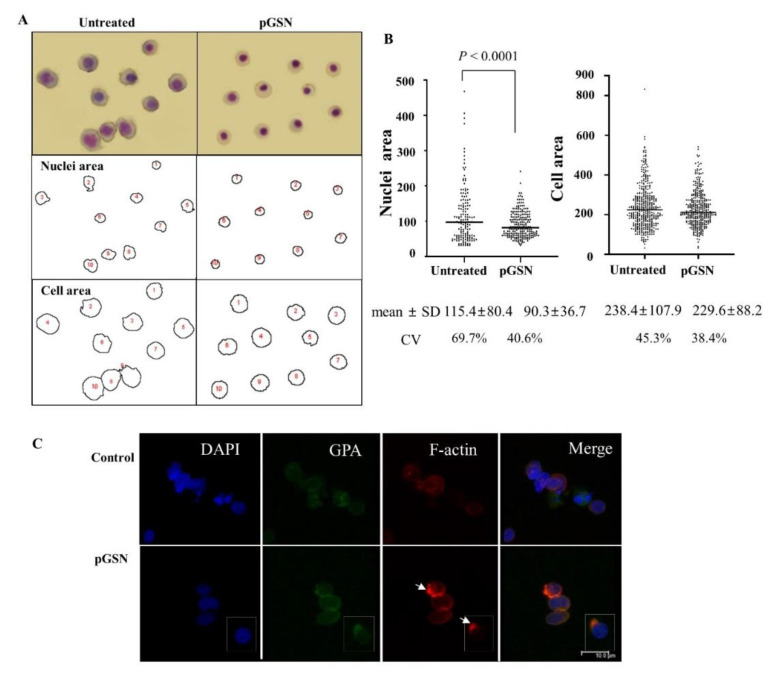
pGSN promotes consistency of nuclear and cytoplasmic sizes. (**A**) The relative surface areas of cells and nuclei were calculated. Representative cell images are shown. (**B**) After treatment with pGSN, the areas of nuclei (untreated; *n* = 151, pGSN; *n* = 210) and cells (untreated; *n* = 416, pGSN; *n* = 382) were measured using Image J and analyzed by unpaired *t* test). The coefficient of variation (CV) was calculated by (standard deviation (SD)/mean) × 100. (**C**) Immunofluorescence imaging of cord blood CD34+ cell-derived polychromatic or orthochromatic erythroblasts at stages prior to enucleation by confocal microscopy. Erythroblasts were stained for glycophorin A (GPA, green), filamentous actin (F-actin) with phalloidin (red), and nuclei with DAPI (blue). Note that F-actin-concentrated spots are co-localized with GPA (arrows). One representative experiment involving ten cells is shown. Scale bar, 10 µm.

Since the actin cytoskeleton is pivotal to the structural integrity of cells, we examined the effect of pGSN on cell size [22]. Irregular shape (poikilocytosis) and variability in the size (anisocytosis) of the cytoplasm and nucleus are key features of dysplastic erythroid cells. These dysplastic features were quantified by measuring the surface areas of cells and nuclei after capturing images on cytospin slides (Figure 2A). In the presence of pGSN, numbers of dysplastic erythroblasts were significantly lower than those in controls, with significantly reduced sizes of cell nuclei (Figure 2A,B). In addition, the lower values of the standard deviation (SD) and the coefficient of variation under pGSN conditions shows more evenly sized nuclei and cell size. To demonstrate intracellular effects via actin skeleton modulation, we evaluated actin regulation, which is one of the major functions of gelsolin. When late stage erythroblasts were labeled with phalloidin for F-actin (Figure 2C), concentrated F-actin spots and F-actin caps are appeared at the opposite side from the nucleus in 50% of observed cells, compared to no marked fluorescence in untreated cells, suggesting that exogenous pGSN might regulate F-actin inside cells, as cGSN does.

### 2.3. pGSN Enhances In Vitro Terminal Maturation of Erythroblasts from MDS Patients

**Figure 3:**
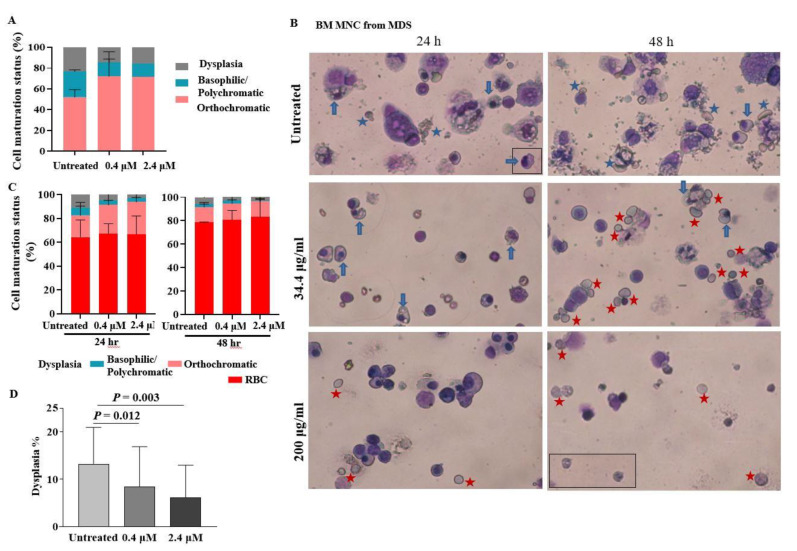
pGSN facilitates the maturation and decreases dysplasia of erythroblasts from myelodysplastic syndrome (MDS) patients. (**A**) Erythroid cells isolated from buffy coats from bone marrow (BM) of MDS patients at diagnosis (case No. 1–2 in Table 2) were treated with pGSN for 24 h. Maturation status was analyzed by counting > 300 cells. (**B**) RBC-depleted BM mononuclear cells (MNCs) derived from MDS patients (case No. 5 in Table 2) were treated with pGSN for 24–48 h. Blue arrows, dysplastic orthochromatic erythroblasts; blue stars, unhealthy RBCs; red stars, intact RBCs. Wright-Giemsa stain, × 200. (**C**) pGSN reduced erythroid dysplasia and increased erythroid maturation and enucleation in a dose-dependent pattern in BM MNCs (*n* = 3; case No. 3–5 in Table 2). (**D**) The percentage of dysplastic cells of the pGSN-treated cells of the five MDS patients in Table 2. The data represent means ± SD.

We investigated whether pGSN would have similar effects on the dysplastic erythroid cells of MDS patients. At first, cells were isolated from BM buffy coat of patients with MDS at diagnosis (Figure 3A). As the buffy coat contains a lot of RBCs and complete removal of RBCs was not possible, assessment of enucleated cells during culture was not available. Therefore, we next collected BM MNCs. When BM MNCs were cultured with pGSN, dysplastic cells, such as nuclear budding, nuclear fragments, multi-nucleation, karyorrhexis, and cytoplasmic vacuolization, were shown more in untreated cells (Figure 3B). Especially, the enucleated erythrocytes in untreated conditions were usually apoptotic showing increased debris and fragile cytoplasm and showed marked anisocytosis (variable cell size) in contrast to the intact, fully hemoglobinized RBCs with similar cell sizes in pGSN treated conditions (Figure 3B). The mean percentage of orthochromatic erythroblasts had increased to 27.4% at the high dose of pGSN compared to 18.2% in the controls at 24 h (Figure 3C). At 48 h of culture, orthochromatic erythroblast portions had slightly decreased compared to that at 24 h, and the mean enucleated RBC count was higher with the high dose of pGSN than that in the control (83.1% vs. 78.5%) (Figure 3C). The dysplasia rates were significantly decreased in all five BM cases in a pGSN dose-dependent pattern (Figure 3D). Taken together, these data suggest that pGSN could enhance in vitro maturation and reduces myelodysplasia of erythroid cells from MDS patients.

### 2.4. GSN mRNA Levels Are Low in PB of Patients with MDS

**Figure 4:**
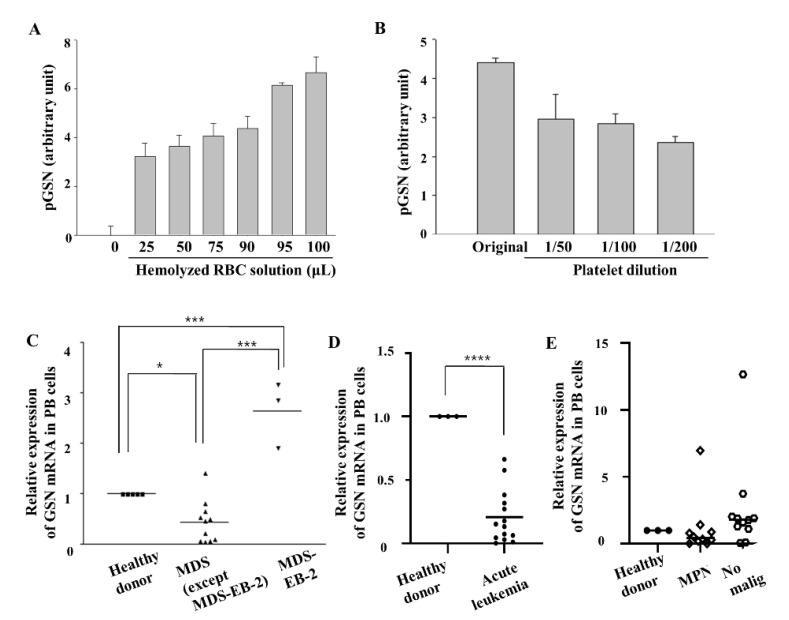
Expression of *GSN* mRNA is reduced in patients with MDS and other hematological malignancy diseases. (**A**,**B**) Hemolyzed RBC supernatants and lysed platelets were diluted to various concentrations, and the levels of pGSN were measured by Enzyme-Linked Immunosorbent Assay (ELISA). (*n* = 3, measured in triplicate). The data are means ± standard error. (**C**) After normalization to *GAPDH*, relative *GSN* mRNA expression levels of peripheral blood (PB) buffy coat cells from 13 MDS patients at initial diagnosis were compared to that of that of healthy donors by room temperature (RT)-qPCR. (**D**,**E**) *GSN* mRNA levels in PB were also measured by RT-qPCR and compared among healthy donors, acute leukemia, myeloproliferative neoplasm (NPM) patients, and the ‘no malig’ group includes other non-oncologic hematology diseases. Levels were normalized to *GAPDH* (* *p* < 0.05, *** *p* < 0.001, **** *p* < 0.0001).

There are no reports describing the level of plasma gelsolin in patients with hemato-oncologic diseases. Therefore, we first evaluated the effects of hemolysis or platelet concentrations on gelsolin protein levels by ELISA or western blotting. However, plasma gelsolin levels could not be accurately measured in BM plasma from hematology-oncology patients, especially those with MDS due to occasional hemolysis that largely affects gelsolin levels (Figure 4A). In addition, measurement of gelsolin protein levels in BM erythroblasts is not easy without flow cytometry sorting in a clinical setting due to high concentrations of gelsolin protein in platelets, which are isolated together with MNCs or buffy coat (Figure 4B). Because platelets are one of the main sources of plasma gelsolin, different platelet concentrations in MDS patients would hamper the analysis of plasma gelsolin levels in nucleated cells.

Therefore, we assessed *GSN* mRNA expression levels, not the protein level, in buffy coat from PB samples of MDS patients with various subtypes (7 males and 6 females). In PB, *GSN* mRNA levels were also significantly low in MDS including single-lineage dysplasia (1 case) or multi-lineage dysplasia (MDS-MLD; 7 cases), and MDS with excess blasts-1 (MDS-EB-1 that is defined by increased myeloblasts of 5–9% in BM; 3 cases), compared to that in healthy (Figure 4C). In contrast, *GSN* mRNA expression in MDS with excess blasts-2 (MDS-EB-2 that is defined by myeloblasts of 10–19% in BM and regarded as a more advanced, pre-leukemic state; 3 cases) showed a significantly increased mean level of 2.85-fold when compared to that in healthy donors, as well as in other MDS cases, except MDS-EB-2 (Figure 4C). The PB *GSN* mRNA levels were significantly correlated with the PB blast percentages (Pearson’s correlation, r = 0.61, *p* = 0.020).

To evaluate specificity of *GSN* levels among other hemato-oncologic diseases, we measured *GSN* levels in more patients at diagnosis (Table 1). *GSN* mRNA expression was significantly decreased in acute leukemia (*t* test, *p* < 0.0001), showing that decreased *GSN* transcription is not specific to MDS subgroups (Figure 4D) and suggesting that the increasing blasts between MDS-EB-2 and de novo acute leukemia might have different pathophysiology in terms of *GSN* expression. Even though platelets secrete gelsolin, the cases with essential thrombocythemia, which has extremely high levels of platelets, showed low levels of *GSN* mRNA, suggesting that platelets in myeloproliferative neoplasms (MPN) might be abnormal regarding gelsolin. In addition, in the MPN group, chronic myelogenous leukemia cases that have very high number of leukocytes, and platelets showed lower expression of *GSN* in three cases, but increased expression in a case with chronic myelogenous leukemia in an accelerated phase that exhibited increased numbers of leukemic blasts like as in MDS-EB-2 (Figure 4E).

From the data of Pellagatti, A. et al. in 2010 and 2013 [23,24], anemic patients were selected by removing the data with Hb > 11.0 g/dL and compared the expression levels of *GSN* mRNA between MDS subgroups. The MDS-EB-2 showed a higher mean level of *GSN* transcript than other MDS subgroups consistent with our data. MDS-EB-2 group especially showed significantly higher *GSN* transcript levels compared MDS with ring sideroblasts (MDS-RS) in Figure S3. In addition, the parameters between BM blast % in the bone marrow and *GSN* transcript values showed a significant correlation with a moderate correlation level (Pearson’s correlation, *r* = 0.22, *p* = 0.007).

### 2.5. pGSN Enhances In Vitro Terminal Erythropoiesis of Erythroblasts in MDS Patients

**Figure 5:**
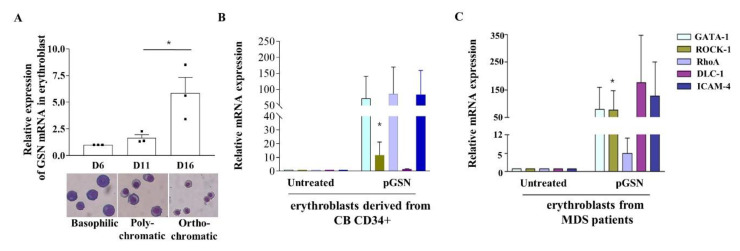
pGSN increases erythropoiesis-related signals in erythroblasts derived from CB and BM of patients with MDS. (**A**) Endogenous *GSN* mRNA was confirmed by RT-qPCR in duplicate, using cultured cells at the indicated days and normalized to the value at culture day 6 (* *p* < 0.05, *n* = 3 cord blood samples). (**B**,**C**) mRNA expression levels of five erythropoiesis- and adhesion-related genes were measured by RT-qPCR in duplicate from CB-derived erythroblasts (**B**) and BM MNCs of MDS patients (**C**) after treatment with pGSN for 24 h. Levels were normalized to *GAPDH* (* *p* < 0.05, Mann–Whitney test; CB-derived erythroblasts, *n* = 4; MDS BM, *n* = 4, cases 2–5 in Table 2). The data are mean ± SEM.

As changes in the expression of *GSN* mRNA during human terminal erythropoiesis were not reported, *GSN* mRNA levels were measured by RT-qPCR at each maturation stage using in vitro cultured erythroblasts derived from CB CD34+ cells. *GSN* mRNA expression was significantly increased up to 5.9-fold from basophilic erythroblasts to orthochromatic erythroblasts, suggesting that *GSN* is involved in erythroblast maturation (Figure 5A).

When the erythroblasts from cord blood and MDS patients were treated with pGSN, *GATA-1* mRNA expression, which increases during erythroblasts maturation and positively regulates erythropoiesis [25], was up-regulated compared to that in untreated controls (Figure 5B,C). We also verified mRNA expression of *ROCK-1*, *RhoA*, *ICAM-4*, and *DLC-1*, the expression of which increases during terminal erythroid maturation and enucleation [26,27,28], and is also involved in actin cytoskeleton organization [29]. Treatment with pGSN markedly increased the expression of these mRNAs in CB-derived erythroblasts (Figure 5B) and up to several hundred-fold in MDS patients (Figure 5C). Especially, *ROCK-1* mRNA that modulates actin cytoskeleton is significantly increased in both CB and MDS groups. These results suggested that pGSN strongly promotes signals for terminal erythropoiesis and cytoskeletal remodeling in late erythroblasts, consistent with the data regarding cell morphology changes.

### 2.6. pGSN Enhances Mitochondrial Polarization in Normal Late Erythroblasts

**Figure 6:**
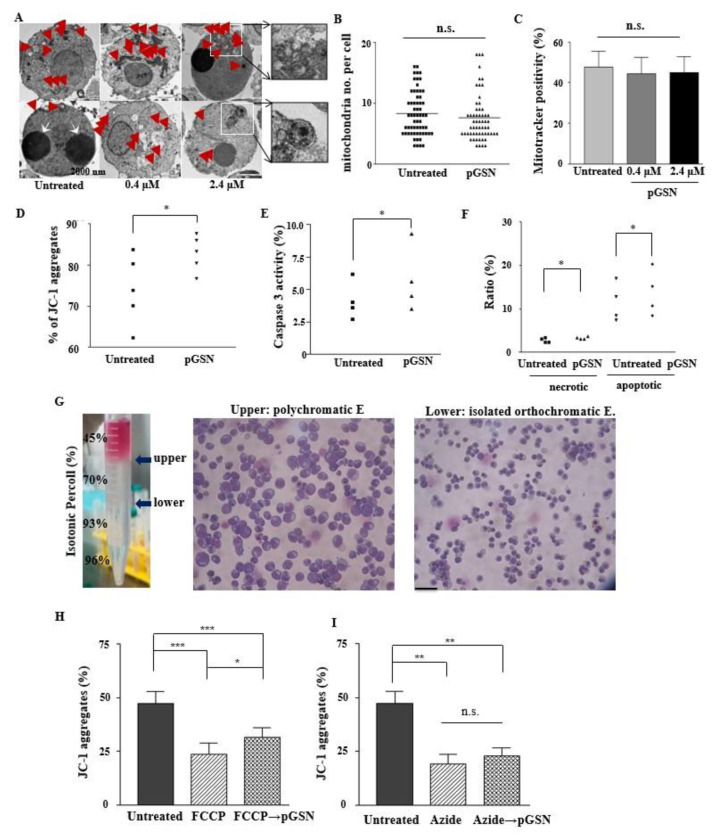
pGSN preserves mitochondrial transmembrane potential in late erythroblasts. (**A**) CB-derived late erythroblasts cultured with pGSN for 24 h were analyzed for mitochondria and mature autophagosomes, compared to the controls, by transmission emission microscopy (TEM). Exocytosis of micro-organelles was observed in cells treated with 2.4 μM pGSN. Representative images of three cases. Red stars, mitochondria; white arrows, nuclei. Scale bar = 2000 nm. (**B**) The numbers of mitochondria per cell were calculated after randomly acquiring TEM images and compared between untreated vs. 2.4 μM pGSN-treated conditions (*n* = 4). (n.s., non-specific.) (**C**) Mitochondrial mass was quantified by flow cytometry using MitoTracker after gating GPA positive cells in CB-derived erythroblasts after treating pGSN for 24 h. (**D**) The mitochondrial membrane potential was assessed in late erythroid cells after treating 2.4 μM pGSN by JC-1 aggregates by flow cytometry. (*n* = 5, * *p* < 0.05) (**E**) Caspase-3 activation was quantified by flow cytometry (*n* = 4, * *p* < 0.05). (**F**) Late stage erythroblasts were analyzed for necrotic (propidium iodide (PI) positive) and apoptotic cells (PI negative/Annexin V positive) by flow cytometry after 24 h of 2.4 μM pGSN treatment. (*n* = 4, * *p* < 0.05). (**G**) CD34+ cell-derived erythroid cells on day 16 of culture were harvested. Then, pure orthochromatic erythroblasts were isolated by gradient centrifugation using various densities of Percoll solutions. Scale bar = 50 µm. (**H**), (**I**) Isolated orthochromatic erythroblasts were incubated with 40 μM FCCP or 10 μM azide for 24 h. After washing, a half of each group was treated with pGSN or vehicle solution for 24 h. Then, all the groups were assessed for JC-1 aggregates (Δψ_m_) by flow cytometry. Data shown are means ± SEM of five independent experiments. * *p* < 0.05, ** *p* < 0.01, *** *p* < 0.001).

To evaluate that in vitro terminal erythropoiesis and dysplastic erythropoiesis which show poor cell viability and the mitochondrial abnormalities were due to insufficient gelsolin, we evaluated the effect of pGSN on mitochondria in CB-derived late stage erythroblasts. When observed under a transmission electron microscope, no marked differences were observed in mitochondrial shape, such as blebbing or swelling, of the outer membrane (Figure 6A). The mean value of the number of mitochondria per cell was similar, regardless of pGSN treatment (Figure 6B). The mitochondrial mass (MitoTracker+/GPA+) was similar among groups (Figure 6C).

As gelsolin was previously reported to block the loss of MMP (Δψm), we measured MMP using a lipophilic cationic dye, JC-1 which selectively enter into mitochondria and reversibly change color from green to red along with increased MMP [30,31]. With the treatment of pGSN, the aggregated form (red fluorescence) of JC-1 was significantly increased which means the MMP is high, demonstrating that pGSN was effective in preserving the MMP, compared to that in the control (Figure 6D). With the addition of pGSN, caspase-3 activity and apoptotic cells were significantly increased, but with very slight differences (Figure 6E,F) which correlates with enhanced maturation #by pGSN [32].

### 2.7. pGSN Preserves and Restores MMP

Erythroid cells are required to maintain MMP before enucleation for energy production [33,34]. We therefore isolated primary orthochromatic erythroblasts and allowed them to mature towards enucleated RBCs in the presence of MMP blockers, which lead to decreased ATP production. The orthochromatic erythroblasts’ MMP was blocked by FCCP (a mitochondrial uncoupler) and sodium azide (a complete mitochondrial respiratory inhibitor). After treatment with each reagent for 24 h, there were significant decreases in JC-1 aggregates in all groups. Then, the subsequent treatment of pGSN for 24 h made JC-1 aggregates (%) reversed significantly in only FCCP-treated cells but not in azide-treated cells, showing that pGSN could normalize decoupling of the mitochondrial membrane but not the complete blocker of respiratory chain (Figure 6H,I).

## 3. Discussion

Here, we demonstrate novel roles of pGSN during human terminal erythropoiesis in improving cell maturation, enucleation, cell size control, and MMP preservation, with decreased erythroid dysplasia. The *GSN* mRNA levels were significantly low in PB from patients with MDS subtypes and other hemato-oncologic diseases except disease conditions with rapidly growing myeloblasts. Furthermore, when BM cells derived from patients with MDS were cultured in the presence of pGSN, the number of dysplastic erythroblasts was decreased, and that of mature cells was markedly increased, suggesting its therapeutic potential for MDS patients.

Elucidation of the mechanisms involved in terminal erythropoiesis is of great interest for research on myelodysplasia [6]. In the BM, erythroid cells are regulated by adjacent macrophages, which provide critical signals and nutrients. Gelsolin has been first identified in macrophages and is involved in the regulation of secretion and endocytosis [35,36]. In in vitro culture, in the absence of macrophages, late stage erythroblasts tend to show dysplasia-like phenomena, such as inadequate cytokinesis, and are irregular with larger cell sizes [17]. Therefore, we hypothesized that, in stromal cell-free cultures, or in pathologic diseases, such as MDS, erythroblasts lack gelsolin and require exogenous supplementation with gelsolin. This hypothesis was also supported by our previous observations that the maturation and enucleation of CB-derived erythroblasts was promoted by co-culture with mesenchymal stem cells [37].

The actin cytoskeleton is crucial for efficient enucleation in orthochromatic erythroblasts [5], and rearrangements of F-actin are striking morphological changes observed during nuclear expulsion [38]. Although obvious mechanisms involving pGSN could not be identified, we were able to show that F-actin organization was more effective in pGSN-treated cells by demonstrating F-actin accumulation and co-localization with GPA, compared to controls, which would have helped to improve the enucleation.

We found that pGSN increased caspase-3 signaling and apoptosis that was probably caused by the well-known function of pGSN acting as a G2M check point in abnormal cells. We speculate that pGSN might be helpful in suppressing the proliferation of activated abnormal apoptotic clones in MDS, but further studies are required.

When pGSN was added to BM cells derived from MDS patients diagnosed with MDS-MLD, MDS-EB-1, or MDS-EB-2, abnormal terminal erythropoiesis was improved, showing increased numbers of late stage erythroblasts and enucleated RBCs, and better-conserved mitochondrial functions. In addition, pGSN could restore the reversible loss of MMP induced by FCCP but not in irreversible loss induced by sodium azide [39,40]. Mitochondrial abnormalities are related in MDS and in vitro culture conditions, especially in terminal erythropoiesis, and the pGSN could support the mitochondrial function by maintaining its MMP. A transient increase in MMP is also known in enucleating cells [33,34].

MDS is a clonal disease and differs from reactive conditions caused by nutritional deficiencies, such as copper deficiency [41,42]. In contrast, cellular dysplasia in culture conditions can be transient and differ from clonal pathologic states. However, a previous report showed that cytokine treatments could reduce apoptosis and ineffective hematopoiesis in MDS patients [43]. Apoptotic deregulation via Bcl-2 is critical in MDS progression [44]. Anti-apoptotic Bcl-2 family members are associated with the voltage-dependent anion channel (VDAC), which is regulated by gelsolin [45]. In addition, gelsolin is known to block actin-dependent VDAC, which could link the pGSN’s effect on actin modulation and MMP [46]. Furthermore, it was recently reported that BM inflammation can lead to clonal blood disorders [47]. These reports suggest that reductions in apoptosis and inflammation after gelsolin treatment might partly block myelodysplasia progression. In addition, the improvement of normal erythropoiesis by supplement of pGSN would decrease burden of abnormal clones and ineffective hematopoiesis, therefore decreasing the chances that drive clonal evolution. More importantly, the most effective medicines for MDS function by inducing epigenetic changes in dysplastic cells [48]. Gelsolin is frequently repressed epigenetically in cancers [49], and the treatment of HDACi increases gelsolin levels in various cancer cells, suggesting that gelsolin may be a therapeutic strategy in those cancer [50,51,52,53]. In addition to these reports, our data suggest that supplementation of pGSN might be a valuable therapeutic strategy in MDS.

The effect of gelsolin on enucleation and erythroid dysplasia correction might result from effects on the G2/M phase of the cell cycle. Due to the similarity between the structural and signaling requirements of erythroblast enucleation and the cytokinesis process, enucleation is regarded as a form of asymmetric cell division [28].

We assessed *GSN* transcript levels other than pGSN for the detecting accuracy by ruling out the effects of hemolyzed sample. Even though *GSN* mRNA levels were decreased in many hemato-oncologic diseases, MDS-EB-2 and chronic myeloid leukemia in an accelerated phase showed increased levels of *GSN*, suggesting that the pathological conditions with rapidly increasing clones might be different with stable conditions in MDS and chronic myeloid leukemia. Anti-apoptotic signal CD47 is decreased in MDS-EB-1 but turned to increase in MDS-EB-2 in association with increasing granulocyte-macrophage progenitors (GMPs) [53]. And, GMP is known to express gelsolin [47]. In addition, in many cancers, gelsolin expression is increased. Therefore, significantly increased GSN expression in MDS-EB-2 might be due to the increasing GMP including myeloblasts.

The blasts between MDS and de novo acute myeloid leukemia (AML) might have different characteristics as there are different subpopulations in blasts. In our Figure S4, the secondary AML case evolved from MDS-EB-1 showed increased *GSN* expression. Other report demonstrated that a four-fold increased expression of *GSN* mRNA in Lin–Sca+Kit- than in Lin–Sca+Kit+, providing an explanation for this differential response to SDF-1 [54]. They also compared that one of the different key proteins between the two blast subgroups were gelsolin, which were increased in Kit- cells. Gelsolin null cells (kit+) display decreased motility compared with their wild-type counterparts. HSCs with low levels of surface c-Kit expression (c-Kit^lo^) and signaling exhibit enhanced self-renewal potential compared with c-Kit^hi^ HSCs [55].

In addition, supplement with pGSN in patients with low plasma gelsolin levels would not increase abnormal myeloblasts, as gelsolin is one of the abundantly existing proteins in normal plasma. However, further studies using more samples and more specific disease subtypes are required to clarify the role of *GSN* expression in pathologic progression.

For the limitations in our study, how pGSN enters erythroid cells requires further evaluation. In addition, we could not completely exclude the case variation of the cord blood because some cases with very good viability and morphology did not show differences in enucleation percentage between control and pGSN groups, suggesting the pGSN can work in some gelsolin depleted conditions. Nonetheless, further studies with more sophisticated conditions to confirm that pGSN treatment improved the abnormal clonal cells, not just non-malignant cells would clear the potential of pGSN as therapeutics.

We searched possible mechanisms that pGSN might indirectly affect erythropoiesis via other extracellular factors without entering cytoplasm. The possible mechanisms were to bind to sphingosine 1-phosphate (S1P), lipopolysaccharide (LPS), or LPA in serum. First, plasma gelsolin modulates cellular responses to S1P [56]. While S1P promotes erythropoiesis [57], pGSN binds S1P and attenuates cellular effects of S1P, which alleviates the possibility that pGSN promotes erythropoiesis via S1P. Second, LPS evoke a maturation block at the late stage of erythropoiesis by completely suppressing erythropoietin’s stimulatory effects and by decreasing endogenous erythropoietin production in vivo [58]. However, this mechanism is unrelated to our in vitro study. Finally, LPA can affect cells via Toll-like receptors (TRLs) or endothelial differentiation gene receptors and can enhance erythropoiesis [20,21]. Depending on the pGSN concentrations, gelsolin could deliver LPA to its receptors or suppresses LPA stimulation. In another study, pGSN interferes with LPA-induced cellular activation in vitro [59]. Therefore, we measured the concentrations of LPA in cultured media supernatant which might be a key modulator for erythropoiesis in conjunction with pGSN. However, LPA was not detected in our media, excluding the possibility that pGSN would indirectly affect erythropoiesis by binding other plasma factors.

In summary, our findings suggest that human terminally matured erythroid cells require gelsolin for adequate maturation and enucleation. pGSN treatment corrected various abnormalities of erythropoiesis in vitro, as well as in erythroblasts of MDS patients, probably via effects on actin filament rearrangement and mitochondria. Our results suggest that pGSN is essential for human terminal erythropoiesis and might be used as therapeutics for patients with MDS.

## 4. Methods

### 4.1. Cell Culture and BM Collection

This research was approved by the Institutional Review Board of Hanyang University Hospital and Hanyang University in Korea (IRB No. HYU-15-113). All experiments were performed in accordance with relevant guidelines and regulations. Cord blood (CB) was collected from healthy pregnant women and BM aspirates were used after written informed consent was obtained. CD34+ cells were isolated from the CB and cultured in serum -free medium (Stemline II, Sigma, Aldrich, St Louis, MO, USA) supplemented with reagents as previously described [60]. Cytokines supplemented in the media were erythropoietin (6 U/mL, R&D Systems, Minneapolis, MN, USA), stem cell factor (100 ng/mL, R&D systems), and interleukin-3 (10 ng/mL; Sigma) for 0–7 days of culture, erythropoietin (3 U/mL) and stem cell factor (50 ng/mL) for 8–13 days, and erythropoietin (2 U/mL) for 14–18 days. The medium was replenished every 2 days. When more than 30% cells had matured to orthochromatic erythroblasts, as evaluated by Wright-Giemsa staining at culture day 15, cells were cultured with or without pGSN. If not commented otherwise, 2.4 μM pGSN was added.

For experiments involving *GSN* levels in patient PB, samples were collected in EDTA tubes. The diagnoses for subtypes of MDS were based on World Health Organization (WHO) classifications of tumors of hematopoietic and lymphoid neoplasia [61]. The buffy coat, a fraction containing nucleated cells, and mononuclear cells (MNCs) were isolated as described earlier [61]. To culture the BM cells, buffy coat was isolated by centrifugation from fresh BM aspirates and washed in phosphate-buffered saline (PBS), or alternatively, BM MNCs were separated by density gradient centrifugation using Ficoll-Paque Plus (1.077 g/L; GE Healthcare, Uppsala, Sweden). Mononuclear cells were cultured in 2D culture plates and cytokines (erythropoietin 3 U/mL) were added to serum-free media (Stemline II hematopoietic stem cell expansion medium, Sigma) supplemented with 150 μg/mL holotransferrin (Sigma), 90 ng/mL ferric nitrate, 30.8 μM vitamin C, 160 μM 1-thioglycerol, 50 μg/mL insulin (Sigma), 4 μM L-glutamine, and 2 μg/mL cholesterol. The medium was changed every 2 days [16,62].

Summary of BM samples derived from MDS patients is given in Table 2. The specific chromosomal analysis of the BM samples of the five patients are shown in Table S1.

### 4.2. Preparation and Purification of pGSN

The cDNA sequence of human plasma gelsolin was cloned into a pET28a plasmid vector with C- and N-terminal 6xHis tags. The plasmid was transformed into *E. coli* Rosetta2 (DE3) pLysS cells. Protein expression, and purification was performed as previously described [16].

### 4.3. pGSN Treatment in Culture

It has been reported that recombinant gelsolin inhibits MMP (mitochondrial membrane potential) loss at 0.4 μM [63] and another report shows that the median gelsolin level in human plasma is approximately 200 μg/mL (2.4 μM) [64]. Therefore, erythroblasts were treated with pGSN at concentrations of 0, 0.4, and 2.4 μM. Cell viability was evaluated by trypan blue staining. The vehicle solution contained 50 mM Tris-HCL, 200 mM KCL, 0.1 mM EDTA, 1 mM dithiothreitol, and 50% glycerol.

### 4.4. Isolation of Orthochromatic Erythroblasts Using Isotonic Percoll Gradient Methods

Erythroid cells differentiated from CB-CD34 positive cells were collected at culture days 16 or 17. To isolate mature orthochromatic erythroblasts, isotonic Percoll (GE Healthcare, Uppsala, Sweden) were diluted to 96%, 93%, 70%, and 45% in 1× PBS. Two mL of the solutions of different densities were then layered in 15 mL falcon tubes, starting with the denser solution at the bottom of the tube. Next, 2 mL of the harvested erythroid cells was loaded on top of the stacked solution and centrifuged at 1200× *g* for 15 min with the breaks off, followed by collection of the gradient fractions from 70% to 93% of Percoll layers. The separated cells were washed in PBS twice and used for serum-free culture with or without pGSN.

### 4.5. Morphological Analysis

Cells were stained with Wright-Giemsa stain (Sigma) using Cytospin (Hanil Science Industrial, Seoul, Korea). Images were taken with a Nikon Eclipse TE2000-U inverted microscope and analyzed by two experts who have years of experience in research of erythropoiesis and a hematopathologist after confirming that the cell distribution is even. Dyserythropoiesis is defined by nucleus budding, multinuclearity, nuclear hyperlobation, severe megaloblastoid changes, severe cytoplasmic vacuolization, and incomplete hemoglobinization. The surface areas of cultured cells and nuclei were measured with Image J software.

### 4.6. Transmission Electron Microscopy

Cultured late stage erythroid cells were treated with pGSN, harvested, fixed, and prepared for transmission electron microscopy (JEM 1011, Jeol, Eching, Germany).

### 4.7. Immunofluorescence Staining

Cells were harvested and labeled with anti-human glycophorin A (GPA)-PE (BD PharMingen, San Diego, CA, USA) antibodies at 4 °C for 20 min. To assess mitochondrial mass, cells were labeled with MitoTracker Deep Red (BD PharMingen) at 37 °C for 20 min. MMP was assessed by flow cytometry after labeling cells with cyanine dye JC-1 (5,5′,6,6′-tetrachloro-1,1′,3,3′-tetraethylbenzimidazolocarbo-cyanine iodide, Thermo Fisher Scientific, Carlsbad, CA, USA). The cells were incubated with 10 μg/mL JC-1 for 20 min at 37 °C in 5% CO_2_ and a humidified environment, washed twice with PBS containing 1% BSA and resuspended in 1% paraformaldehyde. Caspase-3 activity was measured using a CaspGLOW Fluorescein Active Caspase-3 Staining Kit (BioVision, Milpitas, CA, USA). Apoptosis was evaluated by labeling cells with Annexin V (BD Biosciences, San Diego, CA, USA) and propidium iodide (PI, Sigma). Stained cells were analyzed via flow cytometry using an Accuri C6 personal flow cytometer (BD Biosciences).

For immunofluorescence staining, cells were treated as previously described [16] with His-Tag (D3I1O) XP^®^ Rabbit mAb (Alexa Fluor^®^ 647 conjugate; Cell Signaling, Beverly, MA, USA), Alexa Fluor 633–phalloidin (Thermo Fisher Scientific, Waltham, MA, USA), GPA (BioLegend, San Diego, CA, USA), and 4,6-diamidine-2-phenylindole dihydrocloride (DAPI).

### 4.8. Measurement of Gelsolin Protein Levels Affected by RBC Hemolysis and Platelet Contamination

For RBC isolation, PB from healthy donors were collected in EDTA tubes, and plasma was discarded after centrifugation (500× *g*, 5 min, 4 °C). After washing in PBS, RBCs were lysed using RBC lysis buffer (BioLegend, San Diego, CA) at room temperature (RT) for 10 min. Then, the lysed supernatant was separated and diluted for measurement of pGSN by Enzyme-Linked Immunosorbent Assay (ELISA).

For PB platelet isolation, blood samples from healthy donors were collected in sodium citrate tubes are centrifuged at 100× *g* for 15 min at 4 °C After transferring the supernatant into new tubes, platelets were isolated by centrifugation (800× *g*, 15 min, 4 °C). The concentrated platelets were counted and diluted at various concentrations in 100 μL PBS.

### 4.9. Enzyme-Linked Immunosorbent Assay (ELISA)

96-well flat-bottom plates (Corning Falcon, Corning, NY, USA) were coated with gelsolin antibody (1:50; Abnova, Taipei City, Taiwan) for 1 h at RT, before blocking with 10% FBS in PBS for 1 h at RT. After that, the plates were washed 5 times with 0.05% Tween-20 in PBS. Hemolyzed RBC supernatant, lysed platelets, and standard pGSN protein were added to each well and incubated for 2 h at RT. After removal of the supernatant, each well was washed 3 times with 0.05% Tween-20 in PBS. Horseradish peroxidase-conjugated anti-goat secondary antibody (1:100; Jackson Immunoresearch Laboratories, Avondale, PA) was added to the wells. After incubation for 1 hr at RT, the plates were washed 7 times with 0.05% Tween-20 in PBS. The TMB (Tetramethyl benzidine) substrate reagent (BioLegend, San Diego, CA) was added to the plates for 30 min in the dark at RT. Absorbance was measured at 490 nm using a microplate reader (PerKin Elmer, Waltham, MA, USA). The absorbance was normalized to that of PBS.

### 4.10. Real-Time Quantitative Polymerase Chain Reaction (RT-qPCR)

Total RNA was extracted and synthesized to cDNA as previously described [61]. The sequences of the primers were listed in Table S2.

### 4.11. HPLC ESI-LC/MS

Lysophosphatidic acid (LPA) was measured using electrospray-ionization liquid chromatography/mass spectrometry (ESI-LC/MS, TSQ Quantum Ultra EMR, Thermo Fisher Scientific). Purified LPA (Sigma) was used as a standard for quantification of LPA.

### 4.12. Mitochondrial Transmembrane Potential Inhibition

To evaluate the effects of pGSN on the reversal of MMP inhibition before erythroblast enucleation, pure orthochromatic erythroblasts separated using Percoll density gradient centrifugation were treated with 10 μM sodium azide (Sigma-Aldrich) and 40 μM carbonyl cyanide 4-(trifluoromethoxy) phenylhydrazone (FCCP; Sigma-Aldrich) for 24 h. Then, the cells were washed and half of each group was treated with either pGSN 2.4 μM or vehicle solution for 24 h. Then, all the experimental groups were measured for the JC-1 by flow cytometry.

### 4.13. Statistical Analysis

We examined the significance of the treatment effects by *pGSN* on erythroid dysplasia formation where the treatment effects were tested at different concentrations of *pGSN* as 0 (control), 0.4 μM (low) and 2.4 μM (high). It was nonparametrically tested using Friedman’s test at α = 0.05 whether the proportions of dysplasia formation were significantly different by the treatment group or not. Post-hoc pairwise tests were also followed with Tukey’s adjustment as a multiple comparison test. All statistical analyses were performed using SAS 9.4 (SAS Inc., Cary, NC, USA).

## Figures and Tables

**Table 1:** Diseases characteristics.

Diagnosis	No. of Patients
Leukemia Acute myeloid leukemia Acute lymphoblastic leukemia Adult T-cell leukemia T-cell prolymphocytic leukemia Myeloproliferative neoplasm (MPN)	10 2 1 1
Chronic myeloid leukemia (CML)	4
Essential thrombocythemia	2
Polycythemia vera	1
No hematopoietic malignancy	
Aplastic anemia (AA)	4
Thrombotic thrombocytopenia purpura (TTP)	3
Immune thrombocytopenia purpura (ITP)	4

**Table 2:** Clinical characteristics of bone marrow samples used for cell culture.

Case No.	Age (Year)	Sex	Diagnosis	Isolated Sample
1	55	M	MDS with multi lineage dysplasia	Buffy coat
2	78	F	MDS-EB-2	Buffy coat
3	70	M	MDS with multi lineage dysplasia	Mononuclear cells
4	70	M	MDS-EB-1	Mononuclear cells
5	76	M	MDS-EB-1	Mononuclear cells

Bone marrow (BM) samples from myelodysplastic syndrome (MDS) patients were each cultured; derived from buffy coat and mononuclear cells with or without plasma gelsolin protein (pGSN) treatment. Not compared to the healthy control. Abbreviation: EB, excess blasts.

## Supplementary Materials

Supplementary Materials can be found at https://www.mdpi.com/1422-0067/21/19/7132/s1.
