# Supplementary material for: Role of Plasma Gelsolin Protein in the Final Stage of Erythropoiesis and in Correction of Erythroid Dysplasia In Vitro
Source: Int J Mol Sci. 2020 Sep 27;21(19):7132. doi: 10.3390/ijms21197132 (PMC7583768; doi:10.3390/ijms21197132)
Supplement: Supplementary file 1 [file ijms-21-07132-s001.pdf]

International Journal of *Molecular Sciences*

MDPI

Role of Plasma Gelsolin Protein in the Final Stage of Erythropoiesis and in Correction of Erythroid Dysplasia In Vitro

So Yeon Han [1,2], Eun Mi Lee [2], Suyeon Kim [1], Amy M. Kwon [3] and Eun Jung Baek [1,2,*]

[1] Department of Laboratory Medicine, College of Medicine, Hanyang University, Seoul, Republic of Korea; soyeon806@gmail.com (S.Y.H.); rainbow1114@hanmail.net (S.K.)

[2] Department of Translational Medicine, Graduate School of Biomedical Science and Engineering, Hanyang University, Seoul, Republic of Korea; ghlflek@gmail.com

[3] Biostatistical Consulting and Research Laboratory, Medical Research Collaborating Center, Industry-University Cooperation Foundation, Hanyang University, Seoul, Republic of Korea; amykwon@hanyang.ac.kr

* Correspondence: doceunjung@hanyang.ac.kr; Tel: +82-31-560-2485; Fax: +82-31-560-2489

Table S1. Clinical characteristics of bone marrow samples used for cell culture.

Case No.	Chromosomal analysis
1	47,XY,i(17)(q10),+19[4]/48,idem,+13[16]
2	46,XX,del(11)(q23)[11]/47,idem,+8[7]/46,XX[2]
3	46,XY[20]
4	43~45,Y,add(X)(q28),-4,der(5;21)(p10;q10),+der(5;21),-6,-9,add(11)(p15),der (14)t(?;14)((?;p11.2)t(?;4)(?;q12),del(17)(q25),-18,add(18)(q21), +add(19) (q13.3),i(21)(q10),+i(21)[cp20]
5	47,XY,del(1)(p36.1),del(5)(q13),+8[20]

Chromosomal analysis of bone marrow aspirates from myelodysplastic syndrome patients were performed by G-banded karyotyping using standard techniques. Whenever possible, 20 metaphases were analyzed. Chromosomal abnormalities are described according to the International System for Human Cytogenetic Nomenclature.

Table S2. Primer sequences.

Gene	Forward (5′-3′)	Reverse (5′-3′)
GSN	CTTTGCCTGCTCCAACAAGA	CCGTCTCGATGTACCGCTTA
GATA-1	CCAAGCTTCGTGGAACTCTC	CCTGCCCGTTTACTGACAAT
ROCK-1	AGGAAGGCGGACATATTAGTCCCT	AGACGATAGTTGGGTCCCGGC
RhoA	CTCATAGTCTTCAGCAAGGACCAGTT	ATCATTCCGAAGATCCTTCTTATT
DLC-1	AGTGCGTGCAACAAGCGGGT	TCCGGGTAGCTCTCGCGGTT
ICAM-4	CCGGACTAAGCGGGCGCAAA	AGCCACGAACTCCGGGCTCA
GAPDH	GAAGGTGAAGGTCGGAGT	GACAAGCTTCCCGTTCTCAG

Abbreviations: *GSN*, Gelsolin; *GATA-1*, GATA Binding Protein 1; *ROCK-1*, Rho-associated coiled-coil protein kinase 1; *RhoA*, Ras homolog gene family member A; *DLC-1*, Deleted in liver cancer-1; *ICAM-4*, Intercellular Adhesion Molecule 4; *GAPDH*, Glyceraldehyde 3-phosphate dehydrogenase.

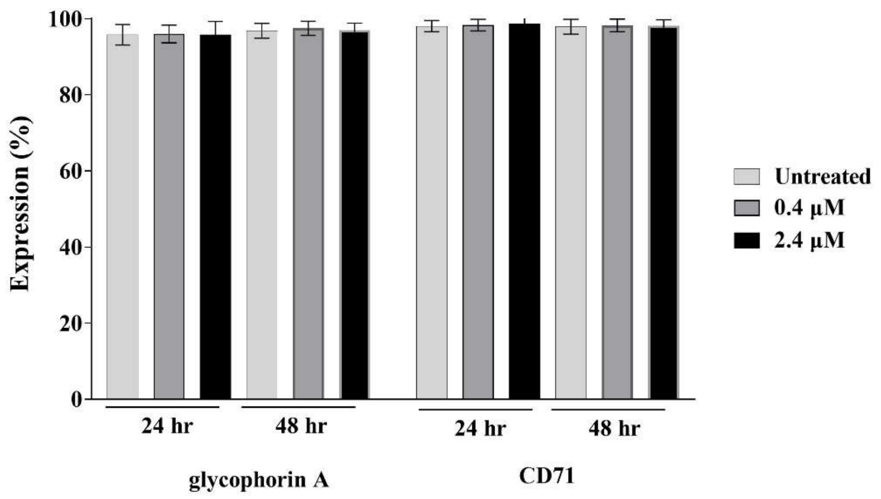

Figure S1. Effect of pGSN on terminal maturation of mature erythroid cells. Erythroid cell specific surface markers were measured by flow cytometry using antibodies against glycophorin A and CD71 at 24 - 48 hr of pGSN treatment in the cells at the polychromatic and orthochromatic erythroblast stages (n = 3). Data are mean ± SD.

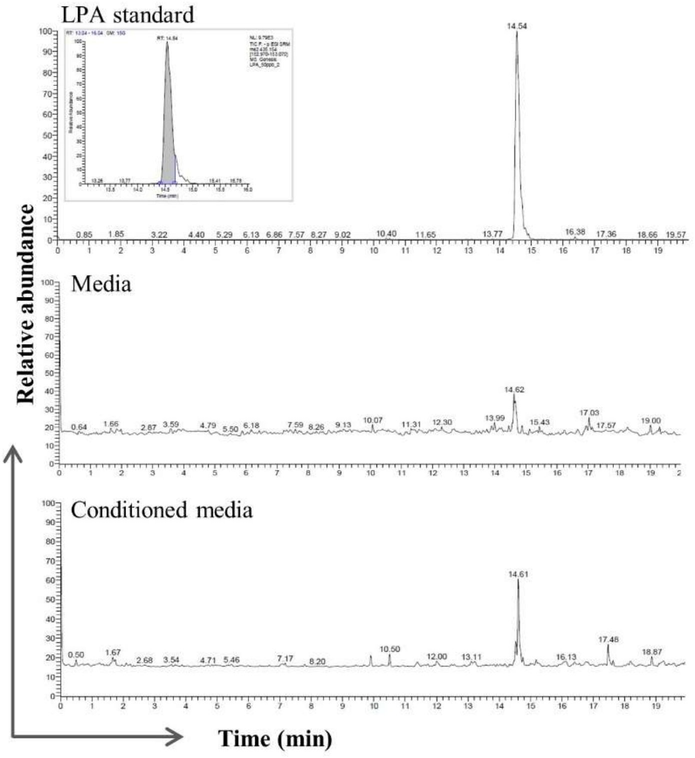

Figure S2. Detection of lysophosphatidic acid (LPA). LPA was measured using electrospray-ionization liquid chromatography/mass spectrometry in the raw media and conditioned media which were media soup harvested after culture of erythroid cells with 2.4 µM pGSN for 24 hr.

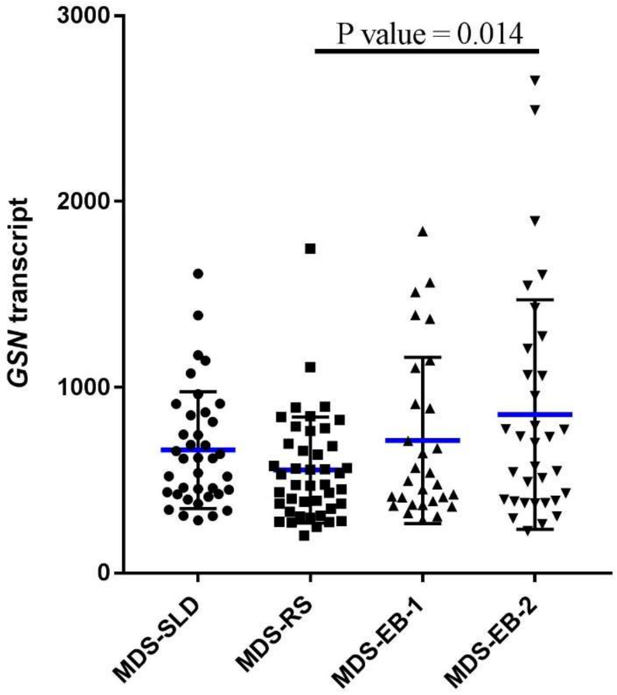

Figure S3. GSN mRNA expression in patients with MDS subgroups. The expression levels of GSN mRNA were compared among MDS subgroups (*P < 0.05, one-way ANOVA, Tukey's multiple comparison test). The lines are mean ± SD.

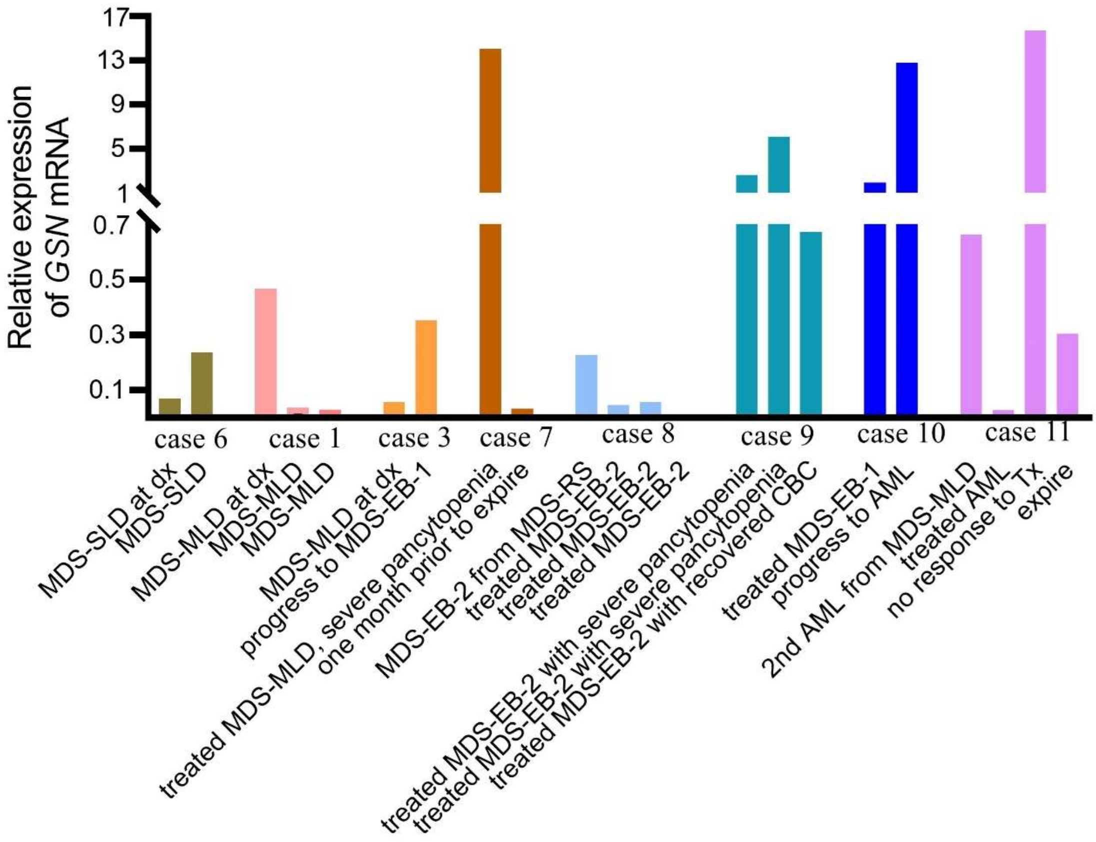

Figure S4. Follow up of PB *GSN* mRNA transcript levels. The *GSN* mRNA expression levels were evaluated for patients with MDS. After normalization to *GAPDH*, relative *GSN* mRNA expression

levels of peripheral blood (PB) buffy coat cells from eight MDS patients normalized to that of that of healthy donors by RT-qPCR were followed up. Cases 1, 3, and 6 are evaluated from diagnosis timing (at dx)', the other samples are acquired after treatment. The same colors mean samples from the same patients. Tx., treatment. The cases usually showed decreased *GSN* level at diagnosis and treatment. The cases with MDS-RS (case 8) which evolved to MDS-EB-2 showed continuously decreased levels, probably related to the disrupted ring sideroblasts. In case 10, the secondary AML progressed from MDS-EB-1 showed increased *GSN* levels, consistent with the increased level in MDS-EB-2, which must be present before worsening to AML. Cases 7 and 11 showed decreased GSN levels near expiration. These data show the increasing tendency of *GSN* mRNA when MDS diseases progress, while *GSN* expression remained low when the patients responded to treatment. .

© 2020 by the authors. Submitted for possible open access publication under the terms and conditions of the Creative Commons Attribution (CC BY) license (http://creativecommons.org/licenses/by/4.0/).
